# Magnetic field effect in natural cryptochrome explored with model compound

**DOI:** 10.1038/s41598-017-10356-4

**Published:** 2017-09-19

**Authors:** Shubhajit Paul, Alexey S. Kiryutin, Jinping Guo, Konstantin L. Ivanov, Jörg Matysik, Alexandra V. Yurkovskaya, Xiaojie Wang

**Affiliations:** 1 Institut für Analytische Chemie, Universität Leipzig, Linnéstr, 3, D-04103 Leipzig, Germany; 2 International Tomography Center, Siberian Branch of the Russian Academy of Science, Institutskaya 3a, Novosibirsk, 630090 Russia; 3 Novosibirsk State University, Pirogova 2, Novosibirsk, 630090 Russia; 4 Department of Chemistry and Biology, College of Science, National University of Defense Technology, 410073 Changsha, China

## Abstract

Many animals sense the Earth’s magnetic-field and use it for navigation. It is proposed that a light-dependent quantum effect in cryptochrome proteins, residing in the retina, allows for such an iron-free spin-chemical compass. The photochemical processes, spin-dynamics and its magnetic field dependence in natural cryptochrome are not fully understood by the *in vivo* and *in vitro* studies. For a deeper insight into these biophysical mechanisms in cryptochrome, we had introduced a flavin-tryptophan dyad (F10T). Here we present the magnetic field dependence of ^1^H photo-CIDNP NMR on F10T and a theoretical model for low-field photo-CIDNP of F10T. This model provides mixing mechanism of energy-levels and spin-dynamics at low magnetic fields. Photo-CIDNP has been observed even at Earth’s magnetic field (~0.05 mT). These experiments prove F10T to be an excellent model compound establishing the key mechanism of avian-magnetoreception and provide insight into the optimal behaviour of cryptochrome at Earth’s magnetic field.

## Introduction

**Figure 1:**
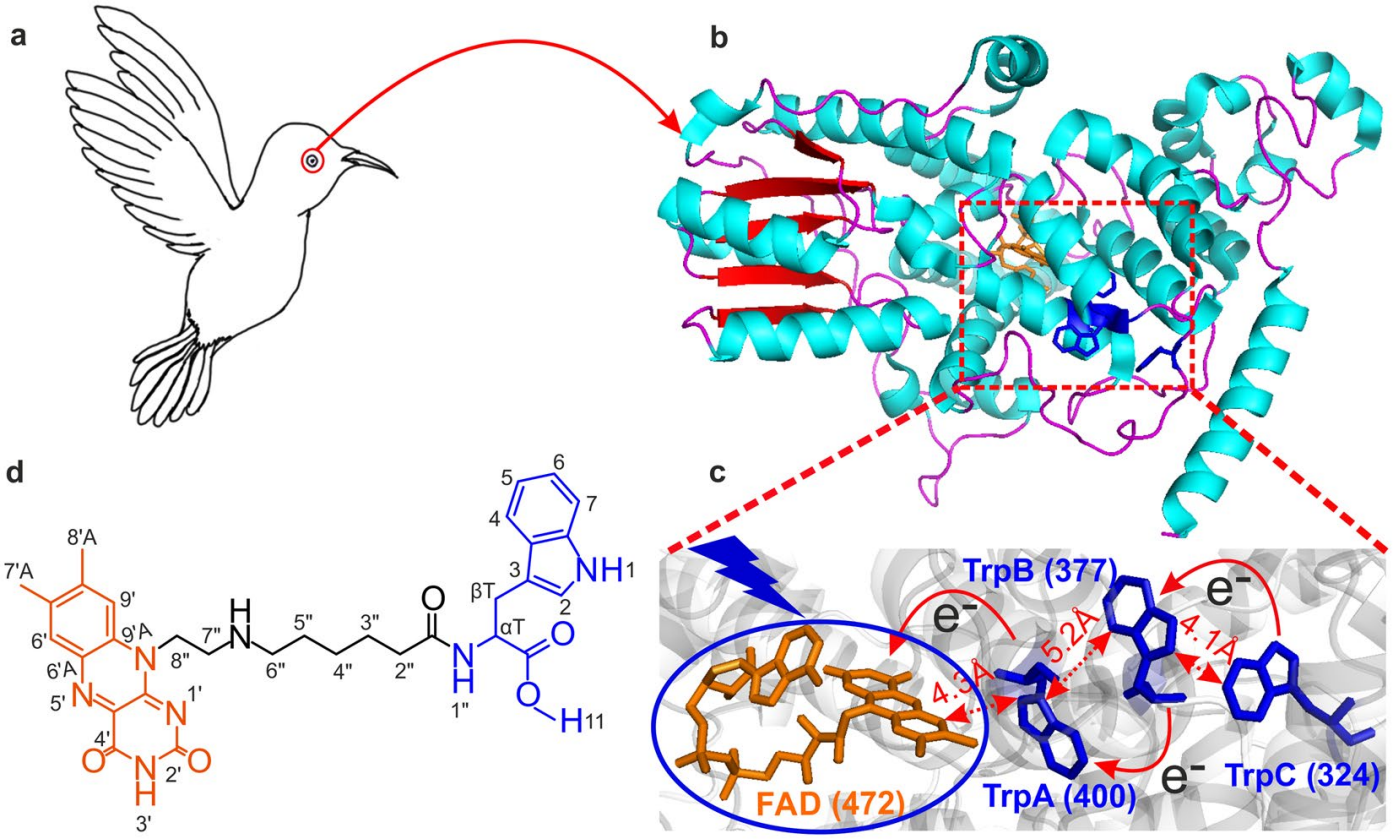
Structural representation of natural cryptochrome and artificial dyad F10T. (**a**,**b**) Migratory bird’s magnetic sense is based on the protein cryptochrome [PDB entry 1U3D]^4^, found in the retina. **(c)** Relative positions of the FAD cofactor and the Trp-triad (TrpA, TrpB and TrpC) of Arabidopsis thaliana cryptochrome. The FAD cofactor is colored in orange and the tryptophans are shown in blue. (**d**) Flavin-tryptophan dyad F10T consisting of a flavin residue (orange) and a tryptophan (blue) connected by an aliphatic linker (black).

Flavins are cofactors in plenty of photo-sensors such as LOV (light-oxygen-voltage) domains, BLUF (Blue-Light Using FAD) proteins, cryptochromes, photolyases (a flavoprotein closely related to cryptochrome) and phototropins^1,2^. Cryptochrome flavoproteins, first found in *Arabidopsis thaliana*, play key roles in growth and development^1,2^. Cryptochromes also control the animal circadian clock and are proposed as magnetoreceptors in migratory birds^1,2^. Recent studies suggested that the origin of such iron-free compass is intervened by a light-dependent quantum effect in the cryptochrome photoreceptor (Fig. 1b and c) that allows migratory birds to sense the Earth’s magnetic field (≈0.05 mT) and to use it for navigation^3–15^. This protein contains a flavin adenin dinucleotide (FAD) cofactor and three tryptophan (Trp) residues (termed TrpA, TrpB, and TrpC, spaced 4–6 Å apart, with a separation of ≈ 18–20 Å between FAD and TrpC) which is interpreted as an electron-transfer chain (Fig. 1c)^1,16^. Upon illumination, the FAD receives an electron from TrpA. Then trpA residue abstracts an electron from the nearby TrpB residue which, in turn, receives one from the third tryptophan, TrpC. As a result of these fast electron-transfer steps, a spin-correlated radical-pair (SCRP) is formed consisting of a flavin anion-radical and tryptophanyl cation-radical on TrpC residue. Schulten^17^ and co-workers first proposed that, in principle, such radical-pair reaction in cryptochrome could act as magnetoreceptor^18,19^ at the Earth’s magnetic field.

The coherent spin–dynamics at low magnetic fields on SCRP^20^ depends on (i) magnitude and distribution of electron-nuclear hyperfine couplings in the two radicals, (ii) the electron-electron exchange interaction and (iii) the dipolar coupling between the two radicals. MFE observed in low polarizing magnetic field (*B*_*p*_) strengths (<5 mT) with phase inversions, is usually referred to as low magnetic field effect or Low-MFE. The exchange interaction, expressed as *J*_*ex*_(*r*) with *r* being the inter-radical separation, has a complex dependence on the electronic properties of the radicals, their separation and the nature of the intervening medium. *J*_*ex*_(*r*) is usually assumed to be an exponentially decaying function (equation (1)) of r and with an adjustable parameter *α*

1 $${J}_{ex}(r)={J}_{0}exp\,(-\alpha r)$$

where *J*_0_ may be either positive or negative.

*J*_*ex*_ was difficult to obtain for natural cryptochrome systems because of experimental limitations and also the low-sensitivity of the previously conducted transient-absorption^21^ and EPR experiments^22^ to the *J*_*ex*_ value. Therefore, till the date, there exists no reliable information about the exchange coupling (*J*_*ex*_) between radical centers in cryptochromes. Previous studies for the Low-MFEs on cryptochrome are completely based on the parameters obtained from bacterial photosynthetic reaction-centre of *Rhodobacter sphaeroides* ^23^. These parameters have no true connection to flavoproteins since the spin-correlated radical-pair contains completely different radicals which possess different photochemical properties and reaction-scheme (reported elswhere^24,25^) compared to that of the flavoproteins. To obtain a precise value of *J*_*ex*_ conquering these experimental constraints, we have intervened a novel approach to the study, i.e. the photo-chemically induced dynamic nuclear polarization (photo-CIDNP) ^1^H NMR studies on cryptochrome model compound in solution-state.

The coherent spin-dynamics in natural cryprochrome allows for a Low-MFE as reported previously in Maeda *et al*.^21^ based upon the studies on *Arabidopsis thaliana* cryptochrome and *Escherichia coli* photolyase^21^. The exact mechanism of ${\rm{singlet}}(S)-{\rm{triplet}}(T)$ interconversion in low magnetic fields, however, remains unclear. Moreover, the precise values of the exchange interaction and recombination rate constant have not been calculated precisely from experimental data-sets obtained with natural cryptochrome or photolyase.

For obtaining the detailed understanding of the Low-MFE on natural cryptochrome, a model compound with similar structural, photophysical and spin-chemical properties as the flavin and TrpC residues in the natural cryptochrome, is required. As first example, a fullerene-based triad model system^26^ has been demonstrated to be able to form SCRP in weak magnetic fields. A directional magnetic field dependence of the kinetics of the SCRP demonstrates phenomenologically that, in principle, a photochemical reaction can act as a magnetic compass. However, the nature of the model system used in these studies is structurally different from that of natural cryptochrome. For these reasons, the magnetic parameters of radical pairs, responsible for magneto-reception in cryptochromes, remain unknown as well as the actual sensitivity of radical pair reactions in cryptochromes to the magnetic field strength and its direction.

Recently, we presented the dyad compound F10T in which a flavin (F) residue is connected to a tryptophan (Trp) residue via an aliphatic chain (Fig. 1d)^27^. The centre-to-centre distance between flavin and tryptophan (~16.5 Å) is close to that between FAD and TrpC (~18–20 Å) in natural cryptochromes (Fig. 1c). The DFT calculations and spectroscopy revealed the presence of different conformations of the model compound depending on the solvent (‘U’- vs ‘bent-Z’- shaped). Solution-state ^1^H photo-CIDNP NMR studies demonstrated the formation of a SCRP in F10T upon irradiation with white-light.

Here we present the time-resolved liquid-state photo-CIDNP ^1^H NMR studies on F10T at high magnetic field (9.4 T) as well as the magnetic field dependence of liquid-state photo-CIDNP ^1^H NMR. Theoretical fitting of field-dependent ^1^H NMR intensities reveals parameters (such as the exchange interaction, molecular mobility parameters, recombination rate constant etc.) that control the spin-dynamics at low magnetic fields (mT) including the Earth’s magnetic field. The importance of our study lies in conquering the lack of knowledge on singlet-triplet interconverion in light-induced SCRP in cryptochrome flavoproteins and computing the parameters for Low-MFE on this SCRP. Furthermore, we aim to connect these parameters to the sensitivity of the chemical processes in the F10T compound to the external magnetic field strength and direction.

## Results

### Time-resolved solution-state ^1^H photo-CIDNP

**Figure 2:**
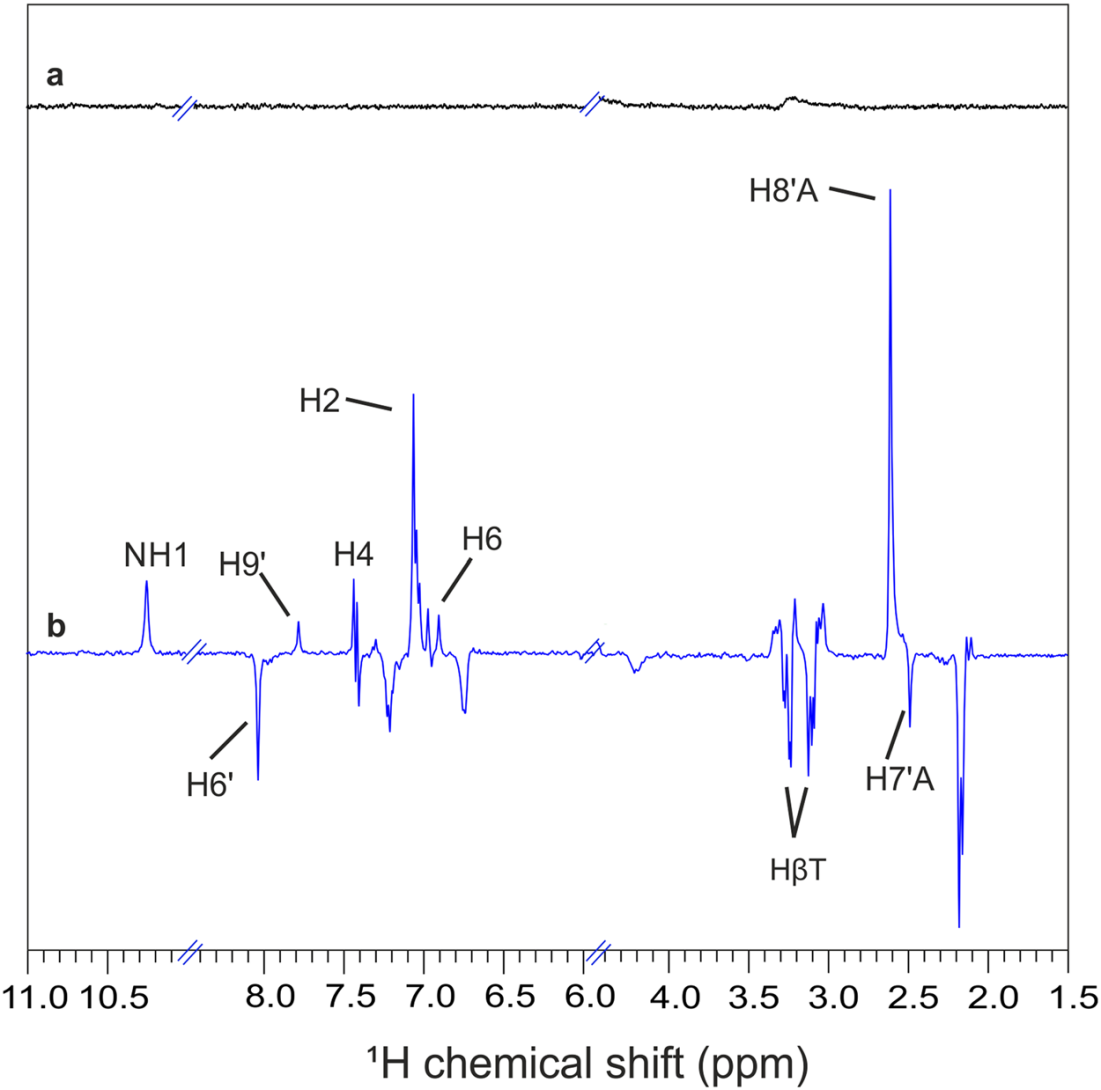
Time-resolved ^1^H photo-CIDNP NMR of F10T. Dark (**a**) and time-resolved ^1^H photo-CIDNP NMR spectra for the illumination with 355 nm 8 ns laser pulse (**b**).

CIDNP spectra were obtained on a 400 MHz NMR spectrometer (Bruker Avance III HD) excited with a 355 nm 8 ns flash Nd:YAG laser (Supplementary Fig. S1). Details of the experimental setup and the pulse-sequence are given in the supplementary information. Dark (Fig. 2, Spectrum a, shown in black) and light spectra (Fig. 2, Spectrum b, shown in blue) which was obtained immediately after laser flash, were recorded at 293 K with the addition of 0.09 mM H_2_O_2_. While no signal occurs in the dark spectrum due to presaturation pulses prior to the laser pulse, several well-resolved absorptive (positive) and emissive (negative) lines are detected under illumination. Light-induced absorptive polarization is observed for the Trp indolyl moiety at positions H2 (7.09 ppm), H4 (7.47 ppm), and H6 (7.03 ppm), while the β-CH_2_ protons (3.29 and 3.12 ppm) are emissively polarized. H6′ (8.06 ppm) and H7′A (2.51 ppm) of the flavin moiety are also emissively polarized, and protons H9′ (7.81 ppm) and H8′A (2.64 ppm) show absorptive polarization. In addition, polarization is observed for the NH1 proton (10.25 ppm) of the Trp moiety. The observed photo-CIDNP effect is due to a photo-induced electron transfer from Trp to flavin forming a SCRP ^T^[F^•**−**^–TrpH^•**+**^] in its pure triplet state as demonstrated by the sign of polarization^28^ for individual protons of flavin and Trp. The photo-CIDNP ^1^H NMR spectrum obtained with F10T shows similar pattern as the spectra of classical liquid-state systems containing free FMN and Trp molecules where the SCRP is also triplet-born (Supplementary Fig. S6)^19,29–33^. Few overlaps of the absorptive and emissive signals have been observed at 7.45 ppm, 3.14 ppm and at 3.29 ppm corresponding to H4 and β-CH_2_ respectively of the Trp residue. This tiny absorptive signal for the β-CH_2_ and emissive signal of the H4 protons are assumed to come from the Trp^•^ radicals from the secondary radical-pairs [F^•**−**^–Trp^•^], formed due to the deprotonation^34^.

No changes have been observed in the photo-CIDNP ^1^H NMR spectra (Supplementary Fig. S5) upon variation of the concentration of F10T in the solution. This suggests that the electron-transfer occurring from F to Trp in the photoexcited molecular triplet state of F10T is essentially intramolecular. In case of free FMN and Trp in solution, an increase in the intensity of CIDNP signal is expected with increasing the concentration. No kinetic evolution on µs timescale (Supplementary Fig. S4) is observed. In case of biradicals (zwitterion), formed due to an intramolecular electron transfer photo-CIDNP ^1^H NMR kinetics are expected on ns timescale^35,36^, whereas in case of intermolecular electron transfer this is on μs timescale^37^. Therefore the electron transfer occurring in F10T is necessarily intramolecular.

### Scheme

**Figure 3:**
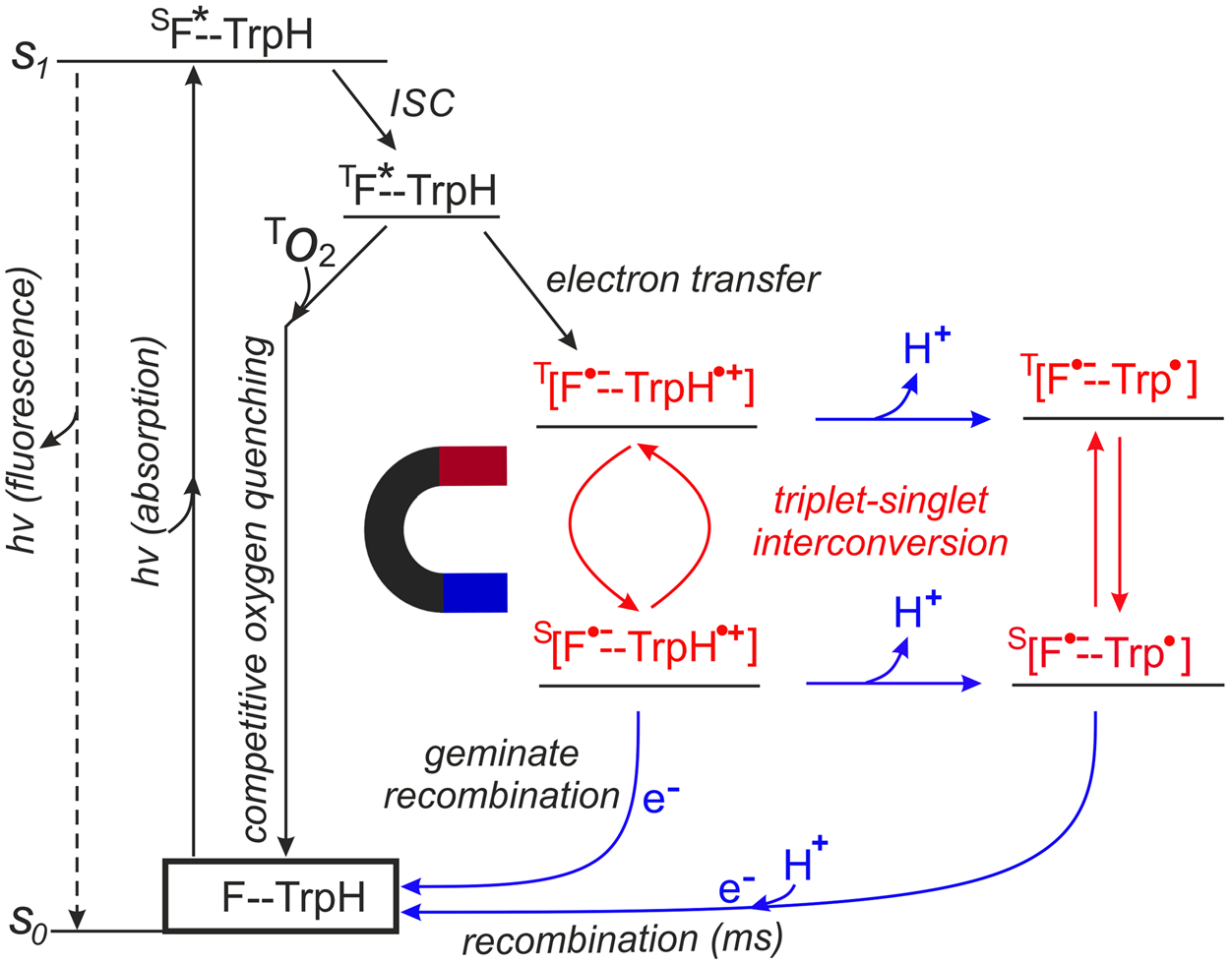
Photo- and spin-chemical reaction scheme for the light-induced reaction-cycle of F10T. Magneto-sensitive stage is highlighted.

Time-resolved ^1^H photo-CIDNP studies lead to the photochemical reaction-scheme for F10T, presented in Fig. 3. Irradiation of the liquid solution of F10T causes an excitation from the electronic ground state (F–TrpH) to the electronically excited singlet state (^S^F*–TrpH). This excited singlet state transforms to the molecular triplet state (^T^F*–TrpH) via intersystem crossing (ISC) in sub-nanosecond timescale. We assume that in the excited molecular triplet state, F10T can have different conformations with dynamic exchange between these geometries in the solution state. Also the free rotations about the single bonds can modulate the orientation between the two terminal moieties in the liquid-state. Subsequently, an electron transfer occurs from the Trp moiety to the excited F (^T^F*), leading to the formation of a SCRP ^T^[F^•−^–TrpH^•+^] which preserves the spin correlation from its triplet precursor on a fast timescale (ps). The short radical-pair lifetime (absence of photo-CIDNP kinetics in μs time-scale and concentration dependence of photo-CIDNP) of F10T implies intramolecular electron-transfer and a formation of a biradical (zwitterion i.e., two radical-centres confined on the same molecule). The SCRP contains a flavosemiquinone anion radical and the tryptophanyl cation radical [F^•−^–TrpH^•+^]. The SCRP is born in its triplet state and partially converts to a singlet state ^S^[F^•−^–TrpH^•+^] via triplet-singlet inter-conversion on a μs timescale. This slow process is presumably because of the strong exchange interaction between the two radical-centres. Due to strong coulomb interaction between the positive and the negative charge-centre (radical-centre), in these energy-states the biradicals obtain a deformed-Z-shaped conformation with reduced separation between the radical-centres compared to the Z-shaped conformation^27^. According to the conservation of spin angular momentum, during the fast electron transfer, the radical pair is formed with the same spin multiplicity as its precursor species. The singlet state of the SCRP ^S^[F^•−^–TrpH^•+^] recombines back to the electronic ground state via geminate spin-allowed recombination on a very fast timescale (ns). Therefore, the kinetics of this pathway is controlled by the triplet-singlet interconversion.

A deprotonation (−H^+^)^21,34^ has been assumed to occur with the primary SCRPs at both triplet ^T^[F^•−^–TrpH^•+^] and singlet ^S^[F^•−^–TrpH^•+^] states upon release of a proton. This deprotonation forms secondary radical-pairs in both triplet ^T^[F^•−^–Trp^•^] and singlet ^S^[F^•−^–Trp^•^] state with a provision of interconversion between these two radical-pairs. Since the strong Coulomb interaction is absent in these secondary radical-pairs, the conformation of the dyad is now predominantly Z-shaped. Because of this conformation, the back-electron transfer followed by the protonation or vice versa, occurs in a longer timescale (ms). This slow process is expressed by the recovery of the electronic ground state.

### Magnetic field-dependence of solution-state photo-CIDNP ^1^H NMR

**Figure 4:**
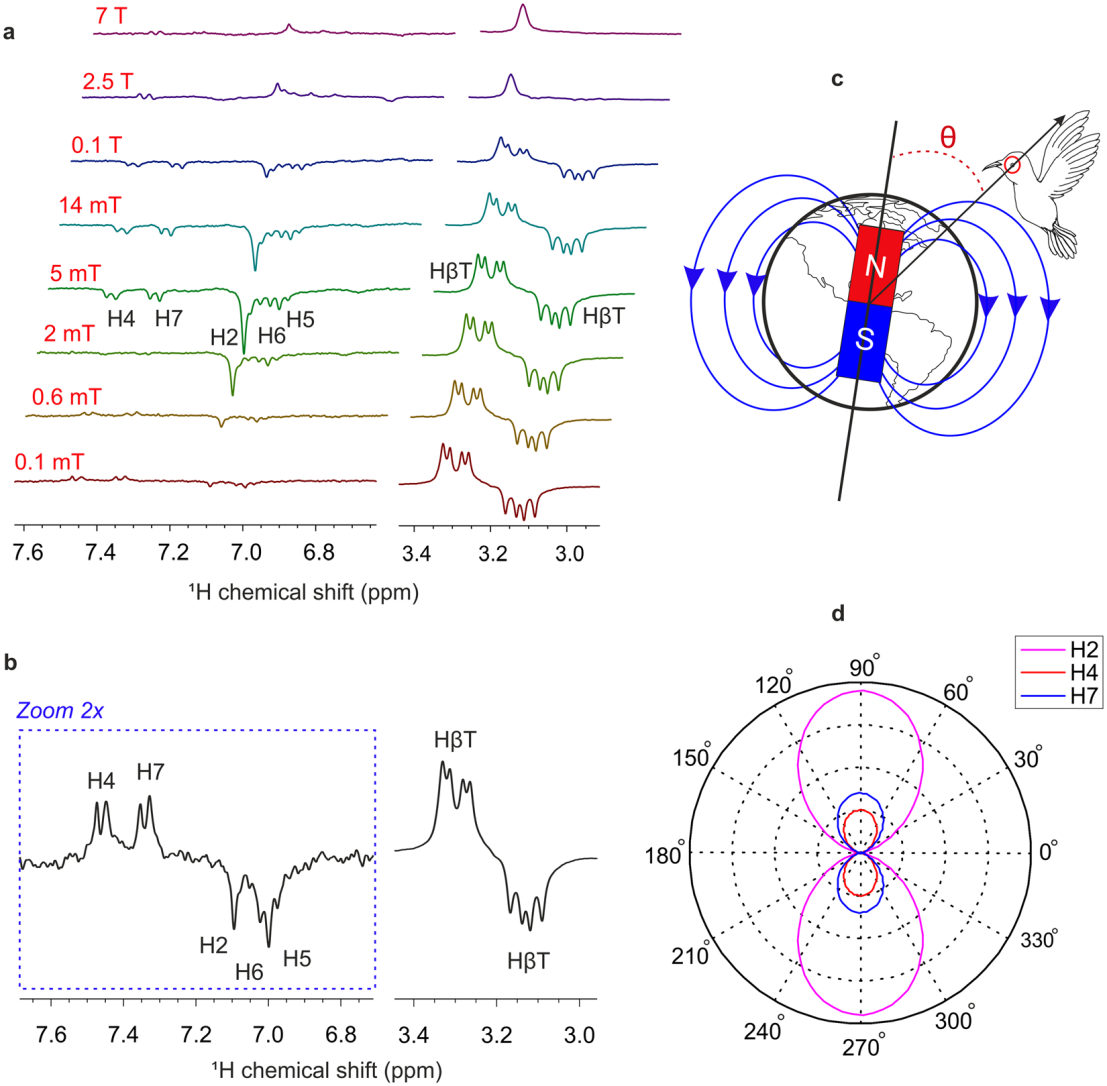
Magnetic field effect on ^1^H photo-CIDNP NMR spectra of F10T. (**a**) ^1^H photo-CIDNP NMR spectra of F10T (in methanol-d_4_) obtained at different polarizing magnetic field (*B*_*P*_) strengths: (i) 0.1 mT, (ii) 0.6 mT, (iii) 2 mT, (iv) 5 mT, (v) 14 mT, (vi) 0.1 T, (vii) 2.5 T, (viii) 7 T. (**b**) ^1^H photo-CIDNP NMR spectra of F10T obtained with the Earth’s magnetic field as the polarizing magnetic field (*B*_*P*_). (**c**) Magnetic force lines of Earth’s magnetic field. (**d**) Proposed variation of ^1^H photo-CIDNP intensities with the change of direction in fully freezed and perfectly aligned F10T dyad. The map is drawn using the software “Corel-Draw” (version x6).

Spectra, obtained for polarizing magnetic fields in the range of 0.1 mT to 7 T, are shown in Fig. 4a. In all cases, the NMR measurements were carried out with field-cycling setup^38^ (Supplementary Fig. S2a) and the acquisitions are done at 300 MHz (7 T) NMR magnet. The pulse-sequence scheme (Supplementary Fig. S2b) is given in the supplementary information. At a polarizing magnetic field strength (*B*_*p*_) of 0.1 mT, emissive polarization for H4 (7.47 ppm), H7 (7.34 ppm) and absorptive polarization for H2 (7.09 ppm), H6 (7.03 ppm) and H5 (7.00 ppm) are observed (Fig. 4a). Amongst the two β-CH_2_ protons (3.29 and 3.12 ppm), one shows absorptive and the other shows emissive polarization. At around 5 mT, all peaks become emissive except of the one of two β-CH_2_ protons. The same emissive signs of these signals suggest that (i) at low magnetic fields the driving mechanism for the triplet−singlet interconversion is not *S* − *T*_0_ spin-sorting but the *S* − *T*_−_ mechanism and (ii) the SCRP in F10T (upon illumination) is due to an intramolecular electron-transfer, forming a biradical (zwitterion). At low magnetic fields, β-CH_2_ protons, having a particular symmetry for their spin-system, are usually long-lived as they become immune to the dipolar interaction which is the main source of relaxation in the low magnetic fields^39^.

### Level Anti-Crossings (LAC) effects in the magnetic field-dependence of photo-CIDNP

At *B*_*p*_ < 2 mT, H7 (7.34 ppm) shows emissive and H5 (7.00 ppm) manifests absorptive polarization and both become fully emissive around a polarizing magnetic field of strength 5 mT (Fig. 4a). However, these two protons do not show any polarization in the photo-CIDNP ^1^H NMR spectrum at high polarizing magnetic field of strength 9.4 T, obtained immediately after the laser pulse (Fig. 2). In low magnetic fields, H5 and H7 get polarization due to polarization-redistribution. This coherent process, mediated by scalar *J*-couplings, is most pronounced around nuclear spin Level Anti-Crossing (LACs)^40–42^. Such a coherent polarization transfer is due to spin coherences formed together with non-equilibrium populations of the spin states. The scalar-coupled spins of the benzene ring of the Trp residue allow H5 and H7 to obtain some polarization from H4 and H6. At *B*_*p*_ of around 5 mT, the photo-CIDNP ^1^H NMR spectrum becomes fully emissive. In this case^22^, the $|S\beta \rangle \rightleftharpoons |{T}_{-}\alpha \rangle$ transitions (where α and β denote nuclear-spin projections) are assumed to be the main channel of triplet−singlet interconversion. In these transitions Z-projection of total (electron plus nuclear) spin is conserved and therefore a flip of one nuclear spin from *α* to *β* state is needed for transition of electron spin from *T*_−_ to *S* ^43^. In these transitions the total electron-nuclear spin is conserved and for the given nucleus the sign of nuclear polarization is independent of the sign of its hyperfine coupling constant. At the low magnetic fields, the photo-CIDNP in biradical with triplet precursor and with negative *J*_*ex*_ recombination from singlet state is formed as a result of transitions from *T*_−_ to *S* electronic state. Most efficiently such transitions occur at LAC (*B*_*max*_ = *J*_*ex*_). Therefore, CIDNP has to have a maximum of emissive signal at the magnetic field corresponding to level crossing.

### Magnetic field dependence of the photo-CIDNP ^1^H NMR

**Figure 5:**
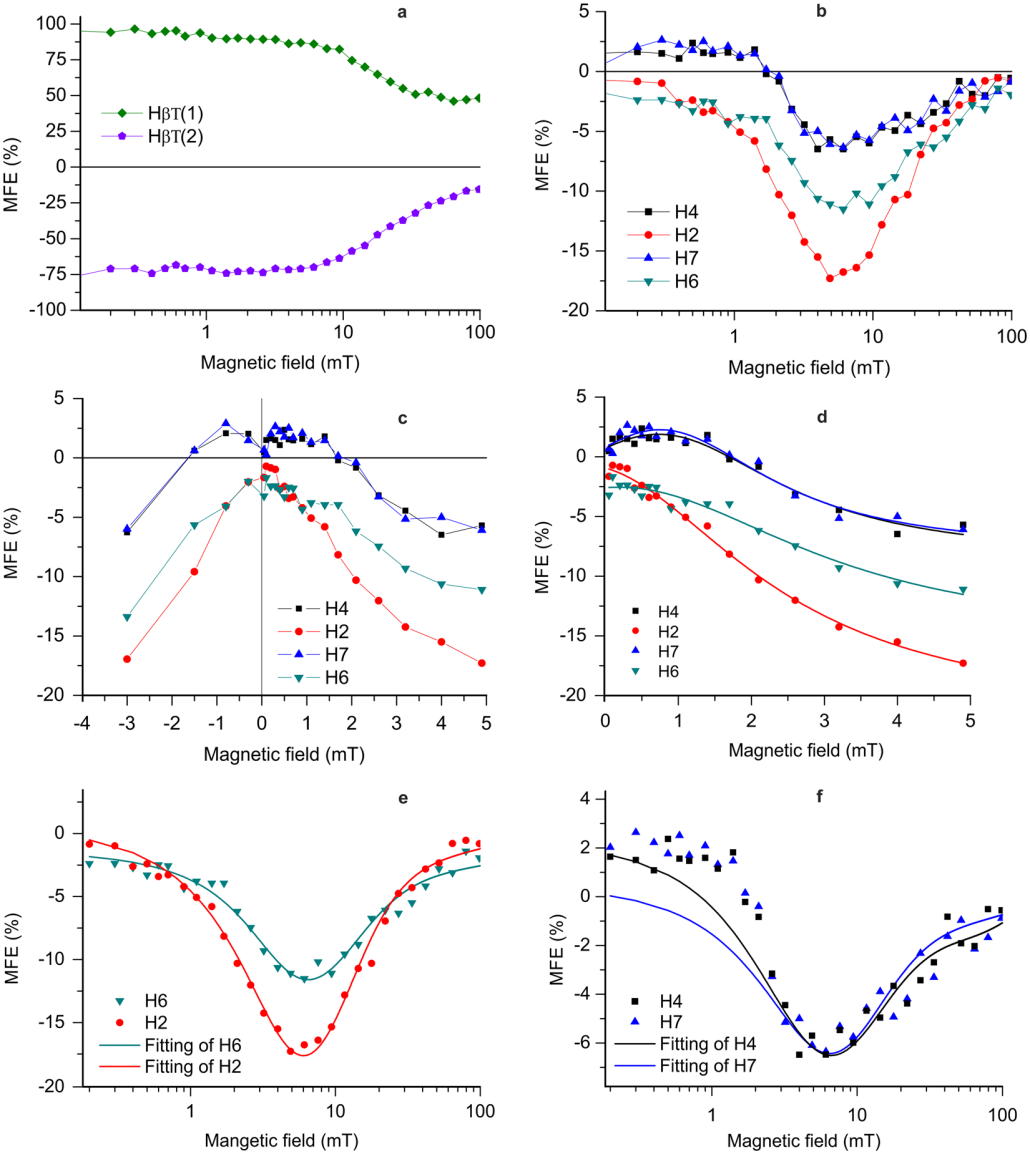
Magnetic field dependence of ^1^H photo-CIDNP of F10T. (**a**,**b**) Normalized intensities plotted as a function of the polarizing magnetic field strengths (*B*_*P*_) for selected ^1^H resonances from F10T for β-CH_2_ protons (**a**), for H2, H4, H6 and H7. (**b**) Low magnetic field effect: normalized intensities plotted as a function of the polarizing magnetic field strengths (*B*_*P*_) for selected ^1^H resonances from F10T dyad for H2, H4, H6, H7 of Trp residue. (**d**) Lorentzian fitting (solid traces) of low magnetic field effect. (**e**,**f**) Theoretical fitting of low magnetic field dependence of photo-CIDNP on F10T in solution state is presented for H2 and H6 (**e**) and **H4** and **H7** (**f**) of Trp residue. Details of the fitting parameters are given in Table 1. Solid lines in (**d**,**e** and **f**) are the fitted curves.

Intensities for different nuclei are presented in Figures 4, 5a and 4, 5b (with X-axis in logarithmic scale) for the β-CH_2_ protons and for H2, H4, H6, H7 respectively. The magnetic field-dependence curve(s) show(s) a dip around the polarizing magnetic field strength of 4 ± 2 mT. We attribute these extrema to the LAC where two energy states (*S* and *T*_−_) avoid their crossing due to a scalar-coupling term in the low magnetic field spin-Hamiltonian^40^. The magnetic field dependence curves are normalized with respect to that of the strongest CIDNP intensities of β-CH_2_ protons which is set to 100% (around *B*_*P*_ = 4.5 ± 2 mT). The maximum negative MFE (on the reaction yield) amongst the protons of the indole ring of the Trp residue is observed for H2 (−17% at *B*_*P*_ = 4.9 ± 0.5 mT). A significant amount of field-effect has also been observed for H4 (−6.7% at *B*_*P*_ = 6.2 ± 0.5 mT), H6 (−11.6% at *B*_*P*_ = 4.0 ± 0.5 mT) and H7 (−6.7% at *B*_*P*_ = 6.2 ± 0.5 mT) protons.

### Sensitivity to extremely low magnetic fields

For *B*_*P*_ > 3 mT, MFEs are consistent with triplet-singlet interconversion induced by hyperfine and Zeeman interactions. However, the sign change in the magnetic field-effect observed mostly for the protons of the indole ring of Trp (H2, H4, H6, H7) below 2–3 mT (Fig. 5c). Such Low-MFE designated by the phase inversions is ~2.9% for F10T. The theoretical fitting of the observed MFE in case of *Arabidopsis thaliana* cryptochrome and *Escherichia coli* photolyase (a flavoprotein closely related to cryptochrome) predicted a magnetic field strength of about 5 mT for the maximum of MFE to be observed^21^. This prediction shows excellent agreement with the MFE observed in case of F10T, with the extremum around 4 ± 2 mT for β-CH_2_ protons and about 5 ± 1 mT for the ring protons (H2, H4, H7, H6) of the indole ring Trp residue (Fig. 5).

Low-MFE is also sometimes characterized by the parameter B_1/2_, i.e., the magnetic field strength at which the value of MFE becomes half of the maximum. From the experimental data, the value of B_1/2_ is obtained to be ~2 mT as shown in Fig. 5d). The observed value of B_1/2_ shows a good agreement with that obtained incorporating the Weller equation^44^ using hyperfine coupling data for lumiflavin radical anion and tryptophanyl radical cation and is approximately 3 mT^21^. However in case of experiments on natural cryptochrome (B_1/2_ ~ 10 mT), a discrepancy between the theory and experiment has been observed and was attributed to the electron-spin decoherence within the radical pair^21^. This fact simply enlightens the importance of a suitable model compound in the study of Low-MFE on natural cryptochrome.

### Theoretical modelling for MFE on ^1^H photo-CIDNP

For a deeper understanding of the evolution of the hyperpolarization and spin-dynamics of F10T under illumination and to elucidate the strength of the interactions effective in the low magnetic fields, (≤100 mT) we use restricted diffusion (RD) model introduced by de Kanter^43^. The spin dynamics of biradical is described using the density matrix *ρ*(*t*), which evolves in time as shown in equation (2)

2 $$\frac{\partial \rho (t)}{\partial t}=-i{H}^{x}\rho (t)+W\rho (t)+K\rho (t)+R\rho (t)$$

where *ρ*(*t*) is a function of the spin-state variables of the biradical and the reaction product and of the distance between the radical centres *r*. The Liouville operator *H* ^*x*^ is related with the spin Hamiltonian, *W* describes the molecular motion and the chemical reactions are expressed in terms of *K*. Matrix *R* is the Redfield relaxation matrix which takes into account two relaxation sources: (i) fluctuating local magnetic fields and (ii) electronic dipole-dipole interaction. The biradical is formed in a certain spin state (S or T) and at a particular distance. To describe the molecular dynamics of the biradical, this model comprises of equilibrium distribution of the end-to-end distance and has been calculated using a special algorithm developed by de Kanter *et al*. In this model, the motion of radical centers is given by diffusion-like motion of the linker. Details of the theory and simulation are given in supplementary information. The fitting of the experimentally obtained magnetic field-dependence of photo-CIDNP for H2, H4, H6 and H7 with RD model has been shown in Fig. 5e,f. The details of the fitting parameters are given in Table 1. *J*_0_ is obtained to be *J*_0_ = −1.6 × 10^9^ mT. With the separation between the two radical centres of 9.14 Å, we obtain the value of the exchange interaction *J*_*ex*_ = −5.12 mT. In addition, the determined value of recombination rate constant is 4 × 10^7^ Hz and that of the effective diffusion coefficient, *D*′, describing the biradical motion is 2.5 × 10^−6^ cm^2^s^−1^. Details of the input parameters, used for simulations, are given in Supplementary Tables S1, S2, S3.

**Table 1:** Parameters obtained from the theoretical fitting of the magnetic field effect on magnetic-field dependent ^1^H photo-CIDNP spectra.

α (Å^−1^)	J_0_ (mT)	D′ (cm^2^s^−1^)	Recombination rate constant (Hz)	*J*_ex_ = *J*_0_ *e* ^−^_*αr*_ (mT)
2.14	−1.6 × 10^9^	2.5 × 10^─6^	4.7 × 10^7^	−5.12

### Earth’s field photo-CIDNP

^1^H NMR spectrum (relevant spectral regions under illumination) has been presented in Fig. 4b. The occurrence of ^1^H photo-CIDNP effect demonstrates that the triplet-singlet interconversion in F10T occurs also at the Earth’s magnetic field. This designates that F10T is in principle able to act as magnetic-field sensor also at Earth’s magnetic field, recognizing itself to be a fully functional and excellent model for natural cryptochromes. Earlier the chemical yield has shown to have a sin^2^(*θ*) orientation dependence^26^. Here *θ* is defined as the angle between the magnetic field vector and the line connecting the position of the bird and the center of Earth’s magnet in a 2D surface, see Fig. 4c. Because of the experimental limitations of NMR, it is not possible (both in solution and solid state) to perform experiments with fully freezed and perfectly aligned compounds. Therefore, for the completeness of the story, we propose that the orientation dependence of ^1^H photo-CIDNP intensities for different protons at Earth’s magnetic field is the same as that for fully freezed and perfectly aligned compound as shown in Fig. 4d.

## Discussion

Here we provide for the first time, a detail study on the spin-dynamics at low as well as high magnetic fields with this model compound. Moreover, this is the largest separation between the two radical centres in a (artificial) biradical to show ^1^H photo-CIDNP and magnetic field dependence of CIDNP in solution-state.

The most important part of the study is the magnetic field-dependence of the quantum yield of the radical-pairs and can be summarized as: (i) A MFE of about +2.9% around 1 mT and about −17% around 4–5 mT (indole ring of the Trp) have been observed along with the strongest for the β-CH_2_ protons (considered as 100%) around *B*_*P*_ = 4.5 ± 2 mT, (ii) ^1^H photo-CIDNP has been observed even at the Earth’s magnetic field (0.05 mT) (iii) B_1/2_ values for different protons of F10T (~2 mT) is in good agreement with that obtained with Weller equation, for Lumiflavin and Trp (~3 mT), (iv) despite of having rotational-diffusion in the solution, F10T shows substantial Low-MFE for polarizing magnetic fields of strength <5 mT. The inclusion of (i) Redfield relaxation theory with two sources of relaxation and (ii) molecular dynamics with Monte-Carlo simulation into the theoretical model for MFE, are completely new to the study of cryptochrome.

Here for the first time we provide the mechanism for triplet-singlet interconversion at low magnetic fields, applicable for the SCRPs of cryptochrome as well. The MFE on the quantum yield of the SCRPs and corresponding polarization pattern of F10T suggests that the triplet-singlet interconversion is controlled mostly by *S* − *T*_−_ mechanism in polarizing magnetic field strengths <100 mT and by *S* − *T*_0_ mechanism in high magnetic fields of several Tesla. Further, this study also provides the values of parameters that control Low-MFE such as exchange interaction *J*_*ex*_ along with *J*_0_ and *α*, effective diffusion coefficient and recombination rate constant. The value of *J*_*ex*_ is obtained to be −5.12 mT and the diffusion coefficient is of the order of 10^−6^ cm^2^s^−1^.

In summary, the experimental difficulties for the studies of Low-MFE on natural cryptochrome systems have been successfully subsided with the ^1^H photo-CIDNP studies on this excellent model compound F10T which provide detailed insight to the behavior and mechanisms of SCRP formation as well as spin-dynamics in low-magnetic fields in case of natural cryptochrome. F10T shows photo-CIDNP even at the Earth’s magnetic field (~0.05 mT). The detailed experimental and theoretical study, presented in this paper, on the F10T model compound at low magnetic fields provides a clear insight into the photo-chemical mechanisms, structure of the SCRP and underlying spin dynamics of natural cryptochrome systems. This breakthrough in understanding of the room-temperature photochemistry and spin-chemistry of natural cryptochromes paves the way towards elucidating the function of flavoproteins and solving the riddle of animal magnetoreception.

## Methods

### Sample preparation

The synthesis details of F10T are given in Paul *et al*.^27^. All the solutions were prepared in methanol-d_4_. A solution of 0.1 mM F10T with 0.07 M H_2_O_2_ has been used for the photo-CIDNP measurements. Details of the experimental setups and pulse sequences used for different ^1^H photo-CIDNP measurements are given in the supplementary information.

### Time-resolved ^1^H photo-CIDNP

Solution-state NMR experiments in were carried out in a Bruker 400 MHz NMR spectrometer using a BBO probe modified to illuminate the sample from side (Supplementary Fig. S1a). A Quantel Brilliant b Nd:YAG laser with a wavelength of 355 nm with a repetition rate of 15 Hz is used as light source. The pulse sequence for time-resolved photo-CIDNP experiments is shown in Supplementary Fig. S1b: bubbling with N_2_ – presaturation (Waltz16)^46,47^ – laser pulse – evolution time – detection pulse – acquisition. The Boltzmann polarizations in the spectrum are suppressed by the presaturation pulses. Therefore, the signals of the polarized products formed due to laser irradiation appear only in the photo-CIDNP spectra.

### Magnetic field dependence of ^1^H photo-CIDNP

Solution-state NMR experiments were carried out at an MSL-300 MHz Bruker NMR spectrometer (Supplementary Fig. S2a) with the detection field of 7 T^38,48^. Field cycling is performed by shuttling the NMR probe-head (home-made) with the sample between positions in space with different magnetic fields. Fields above 0.1 T are controlled by positioning of the sample in the fringe field of the spectrometer cryo-magnet, while fields below 0.1 T are obtained by changing in addition the current in an auxiliary electromagnet. The external field at each position of the probehead is known and controlled with accuracy better than 0.05 mT. A step motor is used to shuttle the sample from the detection field to the variable field in the range from 0.1 mT to 7 T during a time period of less than 0.3 s; the time profile of field variation is precisely mapped. A Quantel Brilliant b Nd:YAG laser with a wavelength of 355 nm with a repetition rate of 15 Hz is used as light source.

A scheme presenting the field-cycling photo-CIDNP experiment, comprising of three consecutive steps, is shown in Supplementary Fig. S2b. In the first step, the sample was kept at low fields (<1 mT) during five times the T_1_-relaxation time to remove thermal polarization. Then the sample was bubbled with nitrogen gas using the above-mentioned technique for 3 s followed by a delay time of 5 s. After that, the sample was irradiated with 20 laser pulses in the polarizing magnetic field Bp. Then the entire probe along with the sample was shuttled to the NMR detection field B_0_. The final step is the application of an RF (π/2) pulse and acquisition.

### Data availability

All relevant data are available from the authors.

## Electronic supplementary material

Supplementary information
