# Supplementary material for: Magnetic field effect in natural cryptochrome explored with model compound
Source: Sci Rep. 2017 Sep 19;7:11892. doi: 10.1038/s41598-017-10356-4 (PMC5605708; doi:10.1038/s41598-017-10356-4)
Supplement: Supplementary file 1 — Supplementary information [file 41598_2017_10356_MOESM1_ESM.pdf]

Supplementary information

Magnetic field effect in natural cryptochrome explored with model compound

Shubhajit Paul[1], **Alexey S. Kiryutin**[2,3], **Jinping Guo**[4], **Konstantin L. Ivanov**[2,3], **Jörg Matysik**[1, *], **Alexandra V. Yurkovskaya**[2,3], **Xiaojie Wang**[4, *]

[1]Institut für Analytische Chemie, Universität Leipzig, Linnéstr. 3, D-04103 Leipzig, Germany
[2]International Tomography Center, Siberian Branch of the Russian Academy of Science, Institutskaya 3a, Novosibirsk, 630090, Russia
[3]Novosibirsk State University, Pirogova 2, Novosibirsk, 630090, Russia
[4]Department of Chemistry and Biology, College of Science, National University of Defense Technology, 410073, Changsha, China

*Jörg Matysik, joerg.matysik@uni-leipzig.de. Tel: +49-341-9736112
*Xiaojie Wang. E-mail: wangxiaojie@nudt.edu.cn. Tel: +86-13308490803

Experimental setup.

Time-resolved solution-state photo-CIDNP ^{1}H NMR experimental setup.

Time-resolved solution-state photo-CIDNP ^{1}H NMR experiments were carried out in a Bruker (Avance III HD) 400 MHz NMR spectrometer using a probe modified to illuminate the sample from side (Supplementary Fig. S1a). The pulse sequence for time-resolved photo-CIDNP experiments is shown in Supplementary Fig. S1b: bubbling with N_2 ─ presaturation (Waltz16)[1,2] ─ laser pulse ─ evolution time ─ detection pulse ─ acquisition. The Boltzmann polarizations in the spectrum are suppressed by the presaturation pulses. Therefore, the signals of the polarized products formed due to laser irradiation appear only in the photo-CIDNP spectra.

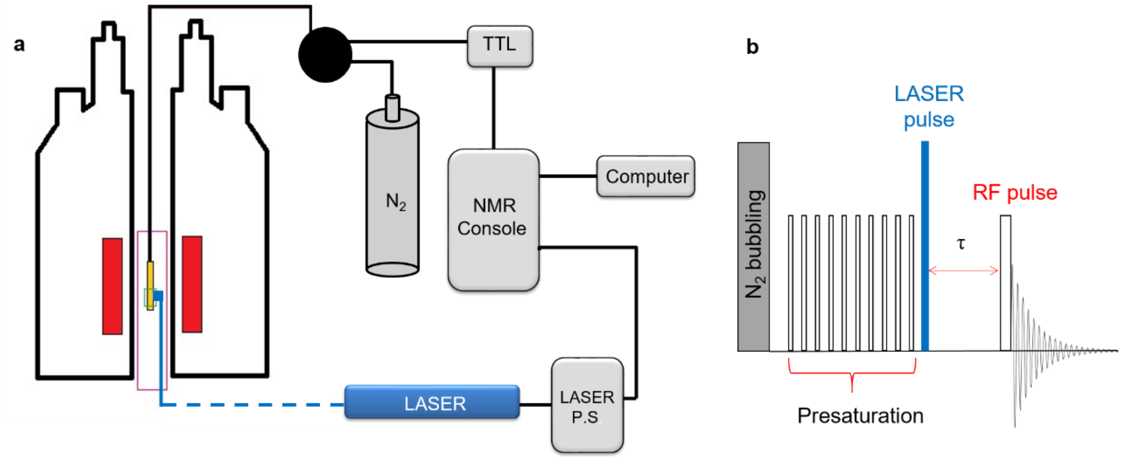

Supplementary Fig. S1: Experimental setup and pulse sequence for time-resolved liquid-state photo-CIDNP ^{1}H NMR. (**a**) Experimental setup and (**b**) pulse-sequence. The pulse-sequence consists of initial bubbling, presaturation pulses, laser pulse, delay (τ) and finally detection (π/2) pulse.

Flash-laser setup.

A Quantel Brilliant b Nd:YAG laser with a wavelength of 355 nm and a repetition rate of 10 Hz is used for initiating the photoreactions which lead to the photo-CIDNP formation. The laser flashes were generated with a pulse length of 6─8 ns and an energy of 80 mJ at the laser output. The pump lamp of the laser was triggered by the triggering impulse from the NMR spectrometer.

Bubbling setup.

At the beginning of each scan the solution was bubbled (pressure ~ 0.3 bar) with nitrogen gas for 3 s followed by a delay time of 5 s to get the solution into a stable condition. In case of time-resolved photo-CIDNP, the sample was bubbled before the pulses for presaturation and in case of field-dependence measurements the bubbling was done in step-1 (Supplementary Fig. S2b). The bubbling was controlled by the spectrometer using a TTL circuit.

Magnetic field dependence of solution-state ^{1}H photo-CIDNP.

All experiments were carried out at an MSL-300 MHz Bruker NMR spectrometer (Supplementary Fig. S2a) with the detection field of ≈ 7 T.[3,4] Field cycling is performed by shuttling the NMR probe (home-made) with the sample between positions in space with different magnetic fields. Fields above 0.1 T are controlled by positioning of the sample in the fringe field of the spectrometer cryo-magnet, while fields below 0.1 T are obtained by changing in addition the current in an auxiliary electromagnet. The external field at each position of the probe is known and controlled with accuracy better than 0.05 mT. A step motor is used to shuttle the sample from the detection field to the variable field in the

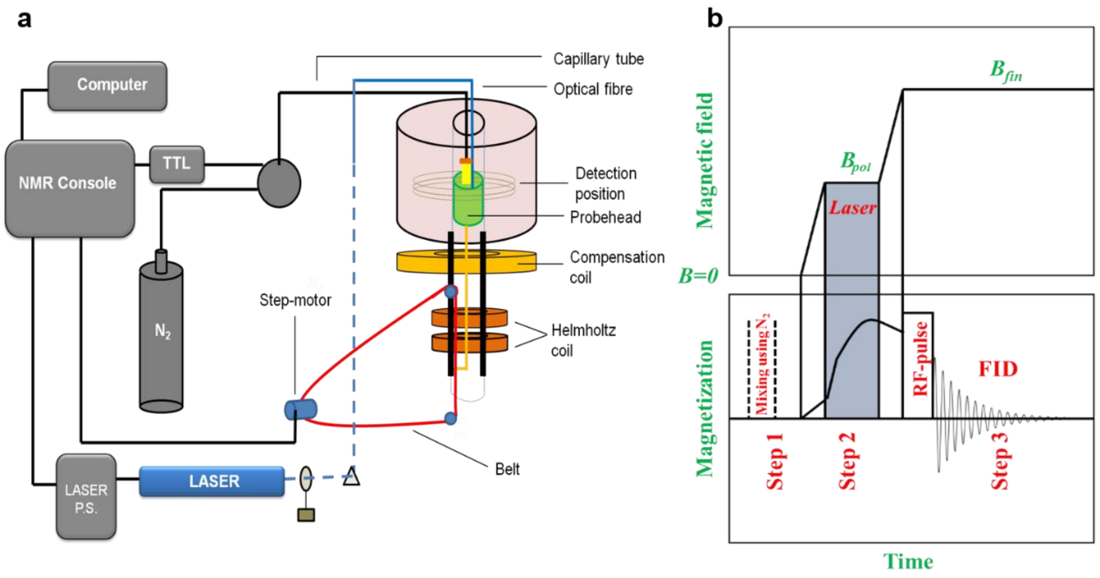

Supplementary Fig. S2. Experimental setup and pulse scheme to measure the magnetic field dependence of ^{1}H solution-state photo-CIDNP with field-cycling: (**a**) experimental setup and (**b**) The pulse-scheme of the light-induced CIDNP experiment with field cycling comprises of three consecutive steps: Step 1: the sample was kept at low fields (< 1 mT) for a time period of five times the T_1-relaxation time to remove thermal polarization, then the sample was bubbled with nitrogen using the above-mentioned technique for 3 s followed by a delay time of 5 s; Step 2: the sample was irradiated with 20 laser pulses (~ 2 s) in the polarizing magnetic field B_p. Then the probe along with the sample was shuttled to the NMR detection field B_0; Step 3: the application of an RF (π/2) pulse and acquisition at the detection field of B_0.

range from 0.1 mT to 7 T during a time period of less than 0.3 s; the time profile of field variation is precisely mapped. A Quantel Brilliant b Nd:YAG laser with a wavelength of 355 nm with a repetition rate of 10 Hz is used as light source.

A scheme presenting the field-cycling photo-CIDNP experiment, comprising of three consecutive steps, is shown in Supplementary Fig. S2b. In the first step, the sample was kept at low fields (< 1 mT) during five times the T_1-relaxation time to remove thermal polarization. Then the sample was bubbled with nitrogen using the above-mentioned technique for 3 s followed by a delay time of 5 s. After that, the sample was irradiated with 20 laser pulses in the polarizing magnetic field B_p. Then the entire probe along with the sample was shuttled to the NMR detection field B_0. The final step is the application of an RF (π/2) pulse and acquisition.

More data on time-resolved ^1H photo-CIDNP of F10T.

^1H photo-CIDNP depletion curves.

To prevent the photo-degradation of the flavin residue,[5] 0.09 mM H_2O_2 was added to the solution of F10T and corresponding depletion curves for different protons of the flavin residue have been presented in Supplementary Fig. S3. Depletion curve is the variation of the intensity (polarization) with number of laser pulses. The depletion curves show that the addition of H_2O_2 (with given molar concentrations) has prevented the photo-bleaching of flavin residue in F10T.

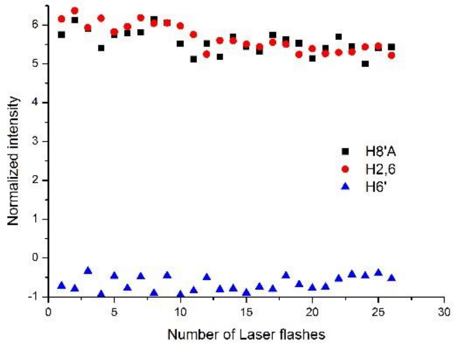

Supplementary Fig. S3. Depletion curves: Normalized intensities are plotted against the number of laser pulses for selected ^1H resonances from F10T (0.1mM in methanol–d_4) with the addition of 0.09 mM H_2O_2.

^1H Photo-CIDNP kinetics.

No kinetic evolution on µs timescale (Supplementary Fig. S4) is observed for any proton from both flavin and Trp moieties. For ^1H photo-CIDNP on biradicals, usually kinetics on ns time-scale is observed.[6] Recording such fast kinetics is beyond the time-resolution of the present photo-CIDNP experimental setup. However, an attempt was made to record the kinetics and the results have been presented in Supplementary Fig. S4.

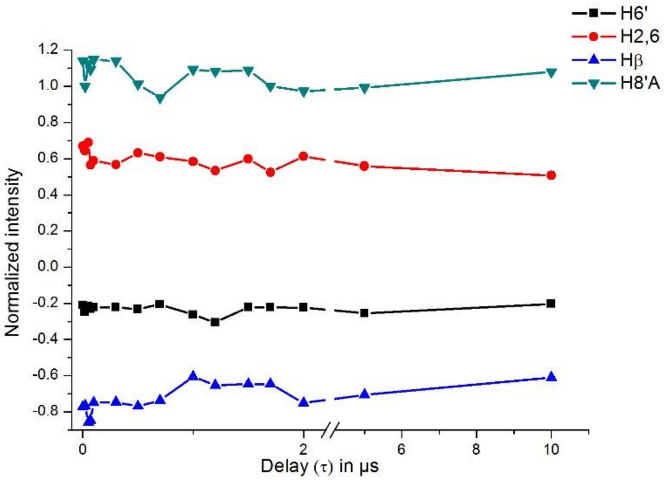

Supplementary Fig. S4. Kinetics: Normalized intensities plotted as a function of the delay-time between the laser pulse and the RF detection pulse for selected ^1H resonances from F10T (0.1mM in methanol–d_4) with the addition of 0.09 mM H_2O_2.

Concentration dependence of ¹H photo-CIDNP.

Upon variation of the concentration of F10T in the solution, no changes have been observed in the photo-CIDNP ¹H NMR spectra (Supplementary Fig. S5). In case of free FMN and Trp in solution, an increase in the intensity of CIDNP signal is expected with increasing the concentration. Hence the photo-CIDNP data demonstrate that the electron transfer in F10T occurs intra-molecularly and therefore, is independent of the concentration.

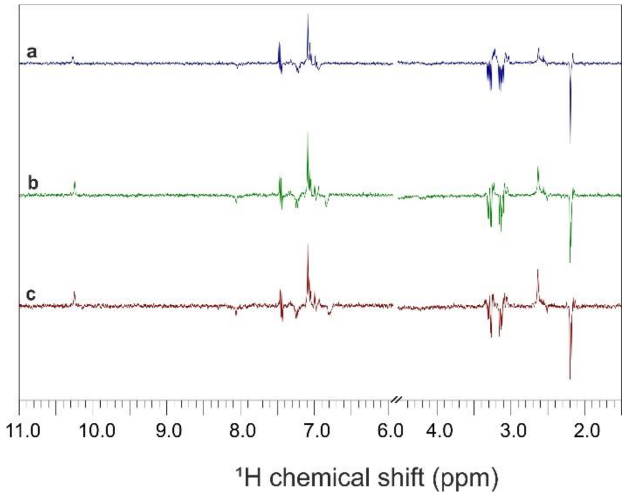

Supplementary Fig. S5. Concentration-dependence of ¹H photo-CIDNP for F10T: ¹H photo-CIDNP NMR spectra of obtained using different concentrations of F10T: (**a**) 0.1 mM, (**b**) 0.2 mM, (**c**) 0.3 mM (in methanol–d4) with the addition of 0.09 mM H_2O_2. All the spectra have been recorded in a magnetic field of ≈ 9.4 Tesla immediately after laser flash.

Photo-chemistry of Flavin-Tryptophan system.

^{1}H photo-CIDNP on flavin mononucleotide (FMN)─Trp systems (added separately in solution).
^{1}H NMR and photo-CIDNP spectra, obtained under continuous illumination using white light from Xenon LAMP for FMN (0.1 mM) ─ Trp (2 mM) in D_2O have been shown in Supplementary Fig. S6. Absorptive polarization has been observed for **H2**, **H4**, **H6** of Trp, while the **β─CH_2** protons of Trp are emissively polarized. For this system, a photo-induced electron transfer from Trp to photo-excited ^{T}FMN forms the tryptophanyl radical cation and flavosemiquinone radical anion:

$$\mathrm{Trp} + {}^{T}\mathrm{FMN} \rightarrow {}^{T}[\mathrm{Trp}^{\bullet+} + \mathrm{FMN}^{\bullet-}]$$

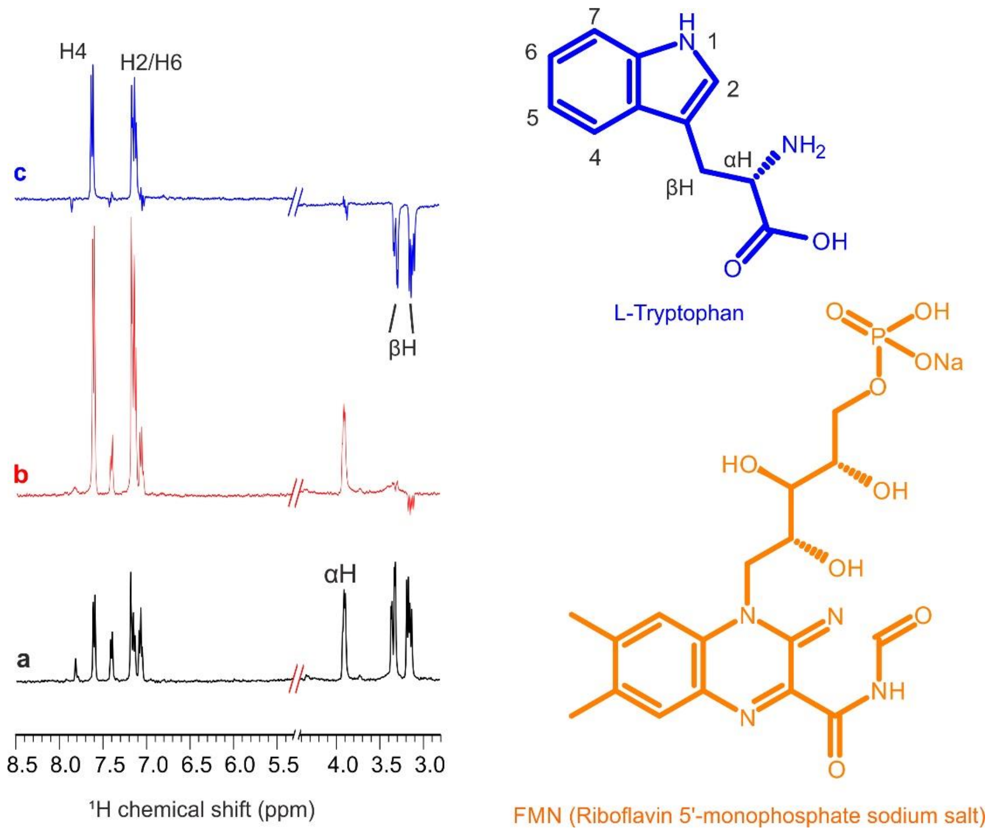

Supplementary Fig. S6. ^{1}H liquid state photo-CIDNP of solution containing FMN (Riboflavin 5'-monophosphate sodium salt) and L-tryptophane in D_2O: (**a**) dark, (**b**) light and (**c**) difference spectra. All the spectra were obtained at DRX-400 MHz NMR magnet and at a temperature of 293K.

Photo-chemistry of flavin.

Flavins are based on the yellow tricyclic isoalloxazine core. They are found widely in nature and the proteins containing flavin are commonly termed as flavoproteins. The three common redox states are the (oxidized) flavin itself (F), the flavosemiquinone radical ($FH^\bullet$) and the flavohydroquinone (FH_2) and their deprotonated forms. Flavins absorb visible light with maximum around 450 nm and 355 nm. Excited triplet flavins can undergo triplet-triplet quenching, quenching by ground state flavins (both inter- and intramolecularly) and quenching by oxygen.

The photochemistry and related reaction scheme for FMN and amino acids such as Trp, Tyr or His (histidine) added separately in solution have been studied widely in last few decades. The observed spin-dynamics is generally explained by the classical radical pair mechanism (RPM)[7,8] in which nuclear-spin interactions control the electron-spin dynamics and therefore the chemical fate of the product formation (Supplementary Fig. S7). Upon illumination, FMN is excited to a molecular triplet state ^{T}FMN* and accepts an electron from the amino acid Trp to yield a geminate radical-pair ^{T}[FMN$^{\bullet-}$ + Trp$^{\bullet+}$] in a pure spin-correlated triplet state. ^{T}[FMN$^{\bullet-}$ + Trp$^{\bullet+}$] forms singlet SCRP ^{S}[FMN$^{\bullet-}$ + Trp$^{\bullet+}$] via interconversion. ^{S}[FMN$^{\bullet-}$ + Trp$^{\bullet+}$] recombines very fast to the ground state via geminate recombination. The triplet SCRP ^{T}[FMN$^{\bullet-}$ + Trp$^{\bullet+}$] recombines back to the ground state in a longer timescale via an intermediate state of spin-uncorrelated free radicals. The frequency of the coherent interconversion of triplet and singlet SCRPs depend on the nuclear spin configurations of the two radicals which are mediated by the electron-nuclear hyperfine interactions.

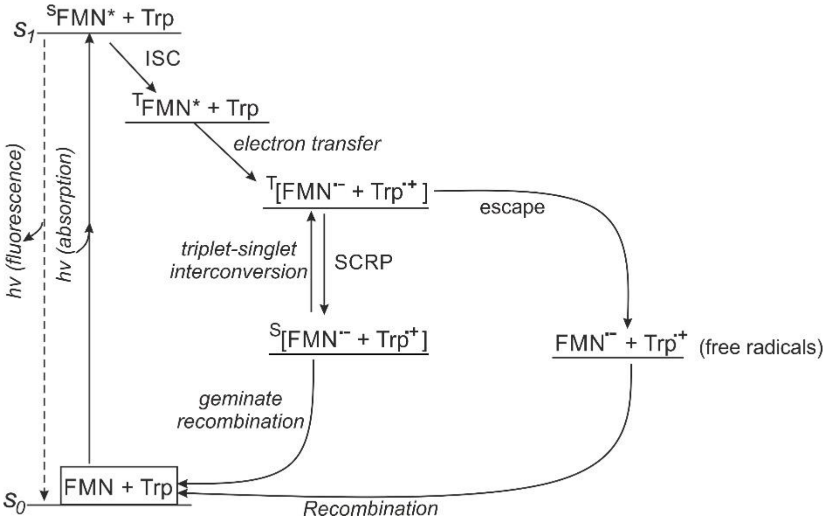

Supplementary Fig. S7. Photochemical reaction scheme of FMN and Trp (added separately in the solution) on the basis of the radical pair mechanism (RPM).

The sign rule of photo-CIDNP.

These observed sign of polarization at high magnetic field in liquid-state photo-CIDNP spectrum can be summarized using the multiplicative sign rule by Kaptein[9] in which the polarization of nucleus n is given by the product of four signs as equation (1)

$$\Gamma_i = \mu \cdot \varepsilon \cdot \Delta g \cdot a_i \quad (1)$$

where a_i is the sign of the hyperfine coupling constant of the ith nucleus, Δg is the sign of the g-value difference $g_m - g_n$, where radical m carries nucleus i. μ is positive for triplet precursor and negative for singlet precursor, while *ε* is positive for recombination products and negative for escape products. The sign of Γ_i determines the sign of the polarization for nucleus *i* whether would be absorptive (positive) or emissive (negative).

Simulation for the theoretical model of MFE on biradical in F10T.

Theory.
To fit the experimentally obtained magnetic field dependence curve for ^1H photo-CIDNP, we have used the theoretical model proposed by de Kanter[10]. In this theory, the spin dynamics of biradical has been described using the density matrix $\rho(t)$, which evolves in time according to equation (2)

$$\frac{\partial\rho(t)}{\partial t} = -iH^x\rho(t) + W\rho(t) + K\rho(t) + R\rho(t) \tag{2}$$

where $\rho(t)$ is a function of the spin state variables of the biradical and the reaction product and of the distance between the radical centres r. Liouville operator H^x is related with the spin Hamiltonian (in angular frequency units) as

$$H^x\rho(t) = H\rho(t) - \rho(t)H \tag{3}$$

Matrix R is the Redfield relaxation matrix and W describes the molecular motion. The chemical reactions are expressed in terms of K. The biradical is formed at time $t = 0$ in a certain spin state (S or T) and at a particular distance.

The spin Hamiltonian of the system can be written as

$$\boldsymbol{H} = \boldsymbol{H^0} + \boldsymbol{H'} \tag{4}$$

where

$$\boldsymbol{H^0} = \frac{1}{2}(g_1 + g_2)\beta\hbar^{-1}B_0(S_{1z} + S_{2z}) + \frac{1}{2}A\boldsymbol{I}.(\boldsymbol{S_1} + \boldsymbol{S_2}) - J(r)(\frac{1}{2} + \boldsymbol{2S_1}.\boldsymbol{S_2}) \tag{5}$$

$$\boldsymbol{H'} = \frac{1}{2}(g_1 - g_2)\beta\hbar^{-1}B_0(S_{1z} - S_{2z}) + \frac{1}{2}A\boldsymbol{I}.(\boldsymbol{S_1} - \boldsymbol{S_2}) \tag{6}$$

g_1 and g_2 are the g-factors of the two radical sites of the biradical. β is the Bohr magneton and B_0 is the magnetic field strength. A is the hyperfine interaction with the nucleus at site 1. The exchange interaction $J(r)$ is expressed as a decaying exponential function of the distance between two radical centers r and with a adjustable parameter α

$$J(r) = J_0\exp(-\alpha r) \tag{7}$$

Using the direct product of the electron spin functions, S, T_+, T_o and T_-, and the nuclear spinfunctions α and β as basis, H^0 is the diagonal and H' is the off-diagonal part of H. Off-diagonal matrix elements in L occur only within two 9 × 9 blocks corresponding to the basis functions $S_\alpha, T_{0\alpha}, T_{+\beta}$ and $S_\beta, T_{0\beta}, T_{-\alpha}$ respectively.

For electron spin relaxation two relaxation mechanisms are considered:

(1) Interactions which are uncorrelated at the two radical sites. These are represented by fluctuating local magnetic fields. They induce singlet-triplet transitions as well as transitions between the triplet levels.

(2) Correlated interactions which induce transitions between the triplet levels only. The most important contribution is given by fluctuations of the electronic dipole-dipole interaction.

The complex relaxation matrix R can be written as

$$R = R_u + R_d \tag{8}$$

Where R_u is the relaxation contribution due to uncorrelated fluctuating local magnetic fields and R_d is the dipole-dipole relaxation term.

For the dynamical behavior of the biradicals the restricted diffusion (RD) model has been taken into account. At first the equilibrium distribution *C(r)* of the end-to-end distance is calculated using a Monte Carlo simulation. The short-range interaction energy of the C and H atoms of CH_2 groups separated by four or less bonds are described by the Buckingham potential. For the long-range interaction energy, which is described by a Lennard-Jones potential, the identities of the individual atoms in the CH_2 groups are ignored.

The normalized distribution *C(r)* is divided into m segments with equal areas (equal probabilities):

$$\int^i C(r)dr = 1/m \tag{9}$$

where $\int^i dr$ denotes the integral over r between the minimum and the maximum values of r in the ith segment. The average distance in segment i is

$$\overline{r_i} = m \int^i rC(r)dr \tag{10}$$

The motion is described by diffusional jumps between the different $\overline{r_i}$'s. Only transitions between neighboring segments are included. By the analogy with classical Brownian motion the transition rates of the jumps are taken as

$$W_{kl} = \frac{D\prime}{(\overline{r_i} - \overline{r_k})^2} \quad \text{for} \quad k = l \pm 1 \tag{11}$$

where D' can be considered as the effective diffusion coefficient for the restricted diffusion.

The chemical reactions of the biradical are represented by the term $K\rho(t)$ in equation (2) and can be written as

$$K = K^c + K^s \tag{12}$$

Where K^c describes the intramolecular coupling or disproportionation reaction and K^s the scavenging reactions. The intramolecular reactions are only possible from the singlet state of the biradical provided it has the conformation with the smallest end-to-end distance (r_d). The intramolecular reaction of the biradical is described by

$$K^c\rho(t) = \frac{-k_p}{2}[O^{S \cdot r_d}\rho(t) + \rho(t)O^{S \cdot r_d}] \tag{13}$$

with k_p the reaction rate constant and $O^{S \cdot r_d}$ the projection operator $|S, r_d >< r_d, S|$ selecting singlet electronic states of the biradical for $r = r_d$. Equation (12) is preferred to the alternative form proposed[11] for reasons of consistency[12].

The scavenging reaction is independent of r and of the electronic state of the biradical. The decay of the biradical density matrix elements is given by

$$K^s\rho(t) = k_s\,\rho(t) \tag{14}$$

where k_s is the scavenging rate constant.

The nuclear spin polarization of the product is given by

$$P = \lim_{t\to\infty}[\rho_{\alpha\alpha p}(t) - \rho_{\beta\beta p}(t)] \tag{15}$$

The solution of the equation of motion (eq. 1) is obtained via the Laplace transformation which yields

$$[sU + iH^x - R - W - K]\rho(s) = \rho^0 \tag{16}$$

with

$$\rho(s) = \int_0^\infty \exp(-st)\,\rho(t)dt \tag{17}$$

where ρ^0 specifies the initial condition and U is the identity matrix. For m conformations (or segments in the RD model) of the biradical, one conformation for the product, and eight spin functions $\rho(s)$ is a column vector with $[8(\mathrm{m}+1)]^2$ elements. Noting that (i) the product occurs only in the S state, (ii) cross terms between different conformations are zero and (iii) of the biradical spin functions 20 elements are important, the relevant elements of $\rho(s)$ are gathered in a $(20\times\mathrm{m}+2)$ column vector. The problem can be reduced using the Hermitian property of ρ. By defining a vector σ:

$$\sigma_{kk} = \rho_{kk}$$

$$\left.\begin{matrix}\sigma_{kl}=(\rho_{kl}+\rho_{lk})/2 \\ \sigma_{kl}=i(\rho_{kl}-\rho_{lk})/2\end{matrix}\right\}\ for\ k < l \tag{18}$$

Equation (14) is reduced to a real form

$$[sU + iL^x - R - W - K]\sigma(s) = \sigma^0 \tag{19}$$

where L^x is obtained by the corresponding rewriting of H^x. The other matrices remain the same. The starting conditions for σ^0 are determined by the spin state of the precursor and by the condition that the biradical is formed in the conformation or segment with the smallest value of r. Assuming no initial electron spin polarization the non-zero elements are for a triplet precursor

$$\sigma_{kkd}{}^0 = 1 \qquad for \quad k = 2, 3, 5, 6, 7, 8 \tag{20}$$

and for a singlet precursor

$$\sigma_{kkd}{}^0 = 1 \qquad for \quad k = 1, 4 \tag{21}$$

The polarization of the product is now given by

$$P = \lim_{s\to 0} s[\sigma_{\alpha\alpha p}(s) - \sigma_{\beta\beta p}(s)] \tag{22}$$

Equation (19) must be solved for gradually smaller values of s until the solution converges. The calculation was performed using a home-written code in FORTRAN.

Simulation parameters.

Supplementary Table S1. Isotropic hyperfine coupling constants (HFCs)* of selected nuclei of tryptophanyl radical cation ($TrpH^{\bullet +}$).

Nuclei	HFC (Gauss)
Hα	–0.93072
Hβ1	16.04575
Hβ2	0.45698
H2	–2.78016
NH1	–4.13008
H4	–4.88005
H7	–3.63695
H6	–2.08282
H5	–0.40011

Supplementary Table S2. g-values* for the two radical centres used in the simulation.

Radical-centre	g-value
$F^{\bullet -}$	2.0034
$TrpH^{\bullet +}$	2.0027

*A. S. Kiryutin *et al.*2007[13]

Supplementary Table S3. Other simulation parameters.

Parameter	Value
B_0 steps	2
Number of steps on field	10000
Number of slices of end-to-end distribution	200
g	8×10^{16} s^{-2}
τ_c	8.0×10^{-10} s
τ_u	1.0×10^{-12} s

References

1 Shaka, A.J., Keeler, J., Frenkiel, T. & Freeman, R. An improved sequence for broadband decoupling: WALTZ-16. *J. Magn. Reson.* **52**, 335─338 (1983).
2 Shaka, A.J., Keeler, J. & Freeman, R. Evaluation of a new broadband decoupling sequence: WALTZ─16. *J. Magn. Reson.* **53**, 313─340 (1983).
3 Kiryutin, A.S., Pravdivtsev, A.N., Ivanov, K.L., Grishin, Y.A., Vieth, H.-M. & Yurkovskaya, A.V. A fast field-cycling device for high-resolution NMR: Design and application to spin relaxation and hyperpolarization experiments. *J. Magn. Reson.* **263**, 79─91 (2016).
4 Grosse, S., Gubaydullin, F., Scheelken, H., Vieth, H.M. & Yurkovskaya, A.V. Field cycling by fast NMR probe transfer: Design and application in field-dependent CIDNP experiments. *Appl. Magn. Reson.* **17**, 211─225 (1999).
5 Maeda, K., Lyon, C., Lopez, J., Cemazar, M., Dobson, C. & Hore, P.J. Improved photo-CIDNP methods for studying protein structure and folding. *J. Biomol. NMR* **16**, 235─244 (2000).
6 Yurkovskaya, A.V., Tsentalovich, Y.P., Lukzen, N.N. & Sagdeev, R.Z. The effett of medium on cidnp kinetics in geminate recombination of biradicals. Experiment and calculation. *Res. Chem. Intermed.* **17**, 145─171 (1992).
7 Mok, K.H. & Hore, P.J. Photo-CIDNP NMR methods for studying protein folding. *Methods* **34**, 75─87 (2004).
8 Hore, J. & Broadhurst, R.W. Photo-CIDNP of biopolymers. *Prog. Nucl. Magn. Reson. Spectrosc.* **25**, 345─402 (1993).
9 Kaptein, R. Simple rules for chemically induced dynamic nuclear polarization. *Chem. Commun.* 732─733 (1971).
10 de Kanter, F. J. J., den Hollander, J. A., Huizer, A. H. & Kaptein, R. Biradical CIDNP and the dynamics of polymethylene chains. *Mol. Phys.* **34**, 857─874 (1977).
11 Freed, J. H., and Pedersen, J. B., 1976, Advances in Magnetic Resonance, Voh 8, edited by J. S. Waugh (Academic Press), p. 1
12 Haberkorn, R. Density matrix description of spin-selective radical pair reactions. M*ol. Phys.* **32,** 1491─1493 (1976).
13 Kiryutin, A. S., Morozova, O. B., Kuhn, L. T., Yurkovskaya, A. V. & Hore, P. J. 1H and 13C Hyperfine Coupling Constants of the Tryptophanyl Cation Radical in Aqueous Solution from Microsecond Time-Resolved CIDNP. *J. Phys. Chem. B* **111,** 11221─11227 (2007).
